# Psychological Interventions Prior to Cancer Surgery: a Review of Reviews

**DOI:** 10.1007/s40140-021-00505-x

**Published:** 2022-01-31

**Authors:** Chloe Grimmett, Nicole Heneka, Suzanne Chambers

**Affiliations:** 1 School of Health Sciences, University of Southampton, Southampton, UK; 2 Faculty of Health, University of Technology Sydney, Sydney, Australia; 3 Faculty of Health Sciences, Australian Catholic University, Brisbane, Australia

**Keywords:** Psychology, Prehabilitation, Health-related quality of life, Cancer, Psycho-oncology, Distress

## Abstract

**Purpose of Review:**

Patients with cancer who have high levels of psychological distress have poor treatment compliance and worse outcomes. This “review of reviews” provides a narrative synthesis of the impact of psychological prehabilitation interventions on individuals awaiting cancer surgery.

**Recent Findings:**

Twenty reviews of prehabilitation with psychological interventions were identified. There is a trend towards improved psychological outcomes following intervention, particularly when psychologist-led. However, there was considerable heterogeneity within interventions, outcome measures, and timing of assessment precluding numeric synthesis. Methodological limitations including non-blinding, absence of stratification, and underpowered studies were also pervasive.

**Summary:**

Providing psychological support early in the cancer pathway and prior to surgery has the potential to improve psychological health and outcomes. The application of existing knowledge in psycho-oncology, including distress screening, is needed in the prehabilitation setting. Consistent outcome assessments, accurate reporting of intervention components and delivery methods, and a consideration of effective systems and economical implementation strategies would facilitate advancements in this field.

**Supplementary Information:**

The online version contains supplementary material available at 10.1007/s40140-021-00505-x.

## Introduction

In 2020, over 19 million new cases of cancer were diagnosed globally [1]. A cancer diagnosis, and subsequent treatment, is a major life stress, giving rise to a range of psychological, physical, social, sexual, spiritual, and daily living difficulties [2]. Approximately, half of people with cancer will experience clinically significant psychological distress related to their diagnosis and treatment [3, 4].

Data from prospective cohort studies show that people with cancer with the highest distress levels have higher death rates compared to those with the lowest levels across several cancer types [5]. Psychological factors, such as depression and anxiety, are associated with worse surgical outcomes [6] such as increased postoperative pain [7, 8], poorer wound healing [9], and increased length of hospital stay [10]. Recent prospective studies also suggest that higher levels of depression and lower levels of self-efficacy to self-manage measured at diagnosis are highly predictive of health-related quality of life 2 years later [11]. How people cope with their cancer diagnosis can also influence decision-making around treatment and future care needs across the cancer continuum [12], and influences long-term psychological morbidity [13, 14].

The last two decades have seen increasing recognition of the importance of integrating the psychological care domain into routine cancer care, culminating in the development of worldwide clinical practice guidelines, quality standards, and the integration of distress as the “6th Vital Sign” (after temperature, blood pressure, pulse, respiration, and pain) [15–17]. It is now well established that psychological care is a critical domain of quality cancer care [18–20]. Imperative within this psychological care is the assessment of all patients to identify their level of distress and psychosocial needs, followed by timely and appropriate referral [15]. This is reflected in global policies and accreditation standards [15] and facilitated through multiple validated screening tools (e.g., NCCN Distress Thermometer [20], Generalised Anxiety Disorder Assessment (GAD-7) [21], and Patient Health Questionnaire (PHQ-9) [22].

While the majority of research and clinical practice has focused on psychological care in the post-treatment/survivorship period, it is increasingly recognized that early targeted psychological interventions, where care is focussed on need, may improve outcomes for people with cancer [23, 24]. The period prior to the start of cancer treatment (prehabilitation) is therefore an ideal point on the cancer continuum to intervene, with opportunities to improve treatment outcomes and reduce the risk of long term psychological morbidity [23, 25].

Historically, prehabilitation has focused on the optimization of physical fitness to improve surgical outcomes. However, research investigating interventions delivered prior to surgery that focus on optimizing psychological health is increasing. The most frequently cited systematic review addressing this issue, published in 2015, included seven studies [26]. There was some suggestion that pre-operative psychological interventions may improve quality of life, but results for core psychological outcomes such as anxiety and depression were inconsistent. Another important consideration is whether exercise-focused and/or multimodal prehabilitation interventions (e.g., combining exercise and psychological support) are more effective at improving psychological health. There is a wealth of literature that describes the therapeutic effect of exercise on anxiety, depression, and quality of life in otherwise healthy populations [27–29], as well as in people with cancer during and after treatment [30–32]; yet to-date, this is less often addressed in the prehabilitation literature.

This paper aims to conduct a “review of reviews” and provide a narrative synthesis of the impact of prehabilitation interventions on psychological outcomes in individuals awaiting cancer surgery.

## Search Strategy

We conducted a meta-review of existing systematic reviews and/or meta-analyses of prehabilitation interventions conducted prior to surgery in cancer care. Reviews were limited to those published in English since 2015. Six databases were searched (October 26, 2020): Web of Science, EMBASE, CINAHL, PsycINFO, PubMed (including MESH terms), and AMED, and additional hand-searches of relevant reference were undertaken. The Preferred Reporting Items for Systematic Reviews and Meta-Analyses (PRISMA) checklist was used to guide review conduct and reporting [33].

A search strategy comprising the domains of prehabilitation, cancer surgery, intervention timing, and publication type was developed and adapted for each database. We acknowledge that this search strategy may not have captured reviews that did not explicitly use the term “prehabilitation” or “preoperative” for interventions targeting people with cancer prior to surgery. However, the term “prehabilitation” reflects common clinical usage and definitions over this review period (2015–2020) [34].

## Results

Thirty-two reviews were initially identified in this meta-review. Eight reviews did not report psychological interventions, psychological-related outcomes, or health-related quality of life (HRQoL) outcomes. Four further reviews noted the prehabilitation interventions included a psychological component (e.g., “anxiety reduction”) but did not report the details or any psychological-related outcomes. Consequently, 20 reviews [26, 35–53] that reported prehabilitation interventions that detailed a specific psychological intervention and/or reported psychological and/or HRQoL outcome measures were included.

Collectively, the 20 reviews reported on 237 studies, with a psychological component to the intervention in 75 studies. Ten of the 75 studies were reported in multiple reviews, leaving 55 individual studies detailing prehabilitation interventions that utilized a dedicated psychological component.

### Characteristics of Prehabilitation Interventions

Over one third (40%, *n* = 8) of the reviews included prehabilitation for multiple cancer types, and the remainder focussed on a specific cancer type: lung (20%, *n* = 4) [35, 46, 48, 49], colorectal (15%, *n* = 3) [36, 38, 39], gastrointestinal (10%, *n* = 2) [37, 53], abdominal (10%, *n* = 2) [42, 44], and laryngeal (5%, *n* = 1) [43] cancers. Prehabilitation interventions were primarily reported in the context of cancer surgery alone (85%, *n* = 17) [26, 35–39, 41••, 42–44, 46–51, 53], with the remaining reviews including prehabilitation prior to neoadjuvant and adjuvant therapy with surgery [40, 45, 52••].

Over half (56%, *n* = 13) of reviews reported multi-modal interventions with various combinations of exercise, nutritional, and psychological components [35–38, 41••, 42, 44, 48–53]. Three (15%) reviews reported psychological interventions only [26, 40, 43], and four (20%) reviews reported psychological and/or HRQoL outcomes following exercise and/or nutrition-based prehabilitation interventions, without a stand-alone psychological component [39, 45–47].

Very few reviews reported the psychological intervention component in detail [26, 40, 41••, 52••]; this was particularly evident for multi-modal interventions. Psychological interventions were categorized using the Framework for Categorising Psychosocial Intervention Components (Table S1) [54]. Reported psychological interventions varied greatly and included the following: education, cognitive-behavioral approaches, relaxation, and supportive counseling (Table S1) [54].

### Cognitive Behavioral Therapy

Interventions were generally delivered by psychologists, psychotherapy trained clinicians and nurses, and research nurses on a one-to-one basis or in groups. However, a number of interventions were self-led with the patient following a home-based program set by a health professional.

Intervention timing, frequency, and intensity were inconsistently reported, if at all. Timing ranged from diagnosis to the day of admission pre-operatively, extending into the recovery period from pre-discharge to 18 weeks post-operatively. Patients received between one and 18 sessions as part of the intervention, lasting 10–90 min. Sessions were generally delivered weekly pre- and post-operatively, with some delivered twice weekly pre-operatively, and others comprised a single session with a health professional followed by patient-directed activities at home (e.g., an audio recording of guided imagery or written education materials). Intervention adherence was also inconsistently reported. Adherence rates ranged from 16–100% including psychological components specifically and the adherence to multimodal interventions.

Overall, the reporting of psychological and HRQoL outcome measures was poor. Often, reviews did not specify all the outcome measures used (Table 1), reporting general outcomes only (e.g., “anxiety”) versus changes in validated measures (e.g., mean scores). The most frequently used psychological measure was the Hospital Anxiety and Depression Scale (HADS), reported in seven reviews [35, 36, 38, 42, 44, 50, 53]. HRQoL measures most often included the Medical Outcomes Study 36-Item Short Form (SF-36); the European Organization for Research and Treatment of Cancer Quality-of-Life Core Questionnaire (EORTC QLQ-C30); and cancer site-specific quality of life measures (Table 1). The specific subscales (e.g., emotional and physical functioning) of HRQoL measures used, such as in the SF-36 or EORTC, were rarely described unless results were significant or showed a positive trend.

**Table 1:** Prehabilitation intervention components, psychological and HRQoL outcome measures reported in reviews

	Intervention components			Psychological Outcome Measures									HRQoL Outcome Measures						
	Exercise ^a^	Nutritional (oral supplement)	Psychological	HADS	POMS	PSE	Atkinson Life Happiness	DES	IES	PKPC	HHI	Psychological (unspecified)	SF-36	EORTC QLQ-C30	EORTC - Other	Cancer Site Specific QoL	EQ-5D	FACT	HRQoL (unspecified)
Chen et al. [40]			✓									✓							✓
Fitzgerald et al. [43]			✓									✓	✓	✓					✓
Tsimopoulou et al. [26]			✓		✓	✓						✓		✓		✓^b^			✓
Daniels et al. [42]	✓	✓	✓	✓									✓	✓		✓^c^			
Schneider et al. [50]	✓	✓	✓	✓									✓						
Piraux et al. [47]	✓	✓	✓										✓	✓	✓^d,e^				
Hijazi et al. [44]	✓	✓	✓	✓									✓						
Boereboom et al. [36]	✓	✓	✓	✓									✓						✓
Bruns et al. [38]	✓	✓	✓	✓									✓	✓					
Chou et al. [41••]	✓		✓				✓						✓	✓		✓^f,g^	✓		
Treanor et al. [52••]	✓		✓		✓			✓	✓										✓
Pouwels et al. [48]	✓		✓							✓	✓								✓
Bolshinsky et al. [37]	✓	✓										✓							✓
Rosero et al. [49]	✓																		✓
Steffens et al. [51]	✓													✓		✓^f,h^			
Vermillion et al. [53]	✓			✓									✓	✓					
Ni et al. [46]	✓												✓						
Garcia et al. [35]	✓			✓									✓	✓				✓^i^	✓
Loughney et al. [45]	✓																	✓^j^	
Bruns et al. [39]		✓											✓	✓					

*DES*, Differentials Emotions Scale; *EORTC QLQ-C30*, European Organisation for Research and Treatment of Cancer Quality-of-Life Core Questionnaire; *EQ-5D*, EuroQol-5D; *FACT*, Functional Assessment of Cancer Therapy; *HADS*, Hospital Anxiety and Depression Scale; *HHI*, Hearth Hope Index; *IES*, Impact of Events Scale; *PKPC*, Power As Knowing Participation In Change Test; *POMS*, Profile of Moods State; *PSE*, Present State Examination; *SF-36*, Medical Outcomes Study 36-Item Short Form

^a^Includes aerobic, resistance, strength, flexibility and/or respiratory; ^b^Prostate Cancer Index (mental); ^c^Gastrointestinal Quality of Life Index (GIQLI); ^d^EORTC BLS-24: Bladder Cancer Superficial; ^e^EORTC BLM-30: Bladder Cancer Muscle Invasive; ^f^International Continence Society “Male” (ICS*male*); ^g^International Prostate Symptom Score (IPSS); ^h^Prostate Cancer Index (mental & physical); ^i^FACT-L: Lung; ^j^FACT-G: General & FACT-BL: Bladder Cancer

### Unimodal Psychological Prehabilitation Interventions

Tsimopoulou et al. (2015) [26] reported a systematic review (*n* = 7) of psychological prehabilitation before surgery (Table S1). This was the only review that identified comprehensively described psychological prehabilitation interventions, timing, session content, and post-operative outcomes measured. While the psychological interventions did not affect surgical outcomes, the authors report that they impacted immunologic function, psychological outcomes, HRQoL, and somatic symptoms. However, the only significant HRQoL change reported was increased physical functioning 1-year post-surgery in a prostate cancer intervention group that received psychologist-led stress management training. Notably, changes in immunological function were only seen in interventions where patients worked individually with a psychologist versus interventions that patients completed primarily at home, e.g., guided imagery audio. Similarly, psychologist-led stress management training, which incorporates problem-solving and coping strategies, appears more effective in terms of psychological (anxiety, depression, distress) and HRQoL (cancer-specific symptoms, physical functioning, fatigue) outcomes compared with audio-recordings of stress management techniques (e.g., relaxation, guided imagery) alone. However, due to small samples and methodological limitations, these results should be interpreted with caution.

A narrative review (*n* = 21) of pre-operative counseling prior to laryngectomy by Fitzgerald et al. [43] noted the systemically poor methodological quality of published studies. The majority (71%, *n* = 15) of papers in this review reported that patients and/or carers considered pre-operative counseling ineffective. This was attributed to a lack of consensus on the definition of pre-operative counseling, counseling provided by health professionals not trained in counseling or psychology, interventions limited to information giving only, and substantial gaps in understanding of pre-operative needs by the patients requiring laryngectomy.

The most recent prehabilitation review of psychological interventions (*n* = 8), conducted by Chen et al. [40], assessed the effectiveness of adjunct psychotherapy prior to cancer treatment, including surgery and/or neoadjuvant and adjuvant therapy. These randomized controlled trials indicated that psychological-based prehabilitation with standard care resulted in better outcomes than standard care alone. Significant results between intervention and control groups included reduced mood disturbances, emotional distress, and anxiety/depression scores and increased physical functioning, immune response, and sleep quality. However, the review did not report specific outcome measures used to determine these results for any of the included trials. Additionally, the authors highlighted notable methodological issues in all papers.

### Multimodal Prehabilitation Interventions with Psychological Components

Multimodal approaches to prehabilitation were reported in 13 reviews. Interventions comprised exercise and psychological approaches (*n* = 7) [41••, 48–53] and exercise, nutrition, and psychological approaches (*n* = 6) [35–38, 42, 44]. Descriptions of the psychological component of multimodal prehabilitation interventions were often poorly, or not at all, reported. Eleven of the 13 reviews reported psychological outcomes as part of the multimodal interventions; however, results were usually only superficially presented and rarely gave details of significant results.

Overall, multimodal prehabilitation interventions which included a psychological component were shown to improve HRQoL. However, this appeared dependent on psychological intervention session intensity, duration, and the number of total sessions [41••, 52••]. Although few reviews identified significant results on psychological outcomes where these outcomes were reported, there was a trend to positive effect. This included lower anxiety, depression, and distress levels and improved mood, functional status, symptom control, immune function, and fatigue.

## Discussion and Observations

This systematic review of reviews identified 32 review papers published since 2015. However, only three of these synthesized data related to psychological interventions and only 17 reviews of multimodal interventions with a psychological component included results relating to psychological outcomes and/or HRQoL.

Psychological care for people with cancer is not yet a consistently included component of prehabilitation research, despite its importance in quality cancer and survivorship care [15, 55, 56]. Existing evidence synthesized in this paper shows a trend towards positive outcomes in this context; however, endemic within this literature are critical methodological shortcomings and heterogeneity limiting the opportunity for evidence synthesis. Psychological interventions either delivered alone or as part of a multimodal intervention are poorly reported with a dearth of details on the “what,” “where,” “when,” “who,” and “how much” related to the interventions delivered. There is significant variability in the outcome measures used and timing of their use, precluding meta-analysis in all the reviews identified. Furthermore, few studies included in these reviews reported data on compliance or evidence of intervention fidelity.

Tsimopoulou et al. [26] suggest that psychologist-led interventions for stress management delivered one-to-one were more effective than home-based self-management such as audio-recording instruction. While intuitive, such conclusions should be interpreted with caution given the lack of compliance data. It is not possible to know if these interventions were indeed ineffective or if participants simply did not engage with them as intended.

There is strong evidence in the survivorship literature of the positive impact of exercise on psychological outcomes in people with cancer [32, 57, 58]. It is intuitive that similar effects would be seen in the pre-operative period. In addition to the trend towards improvements in HRQoL following exercise prehabilitation interventions identified here, qualitative evidence supports this hypothesis. Patients have described that participation in such programs provides them with a sense of control and empowerment [59]. However, definitive conclusions cannot be drawn without a consistent approach to the measurement and reporting of psychological outcomes following unimodal and multimodal prehabilitation interventions.

Common among the psychological interventions included within the reviews was a “one size fits all” approach. There was no evidence that interventions targeted those with heightened distress and/or suboptimal psychological health. It is, therefore, possible that results are subject to ceiling effects. Published guidance for prehabilitation within the management and support of people with cancer advocates for a needs-based prescription [60••], screening patients as close to the point of diagnosis as possible and regularly thereafter as part of survivorship care [56]. Providing personalized and tailored support can maximize impact when resources are limited. Examples of such an approach are beginning to emerge. Van Rooijen et al. [61••] describe a randomized controlled trial of multimodal prehabilitation in colorectal cancer patients. They will offer all patients psychological support in the form of instructions for relaxation and breathing exercises. Furthermore, patients will be screened using GAD-7 and the PHQ-9, a measure of depression. Those identified as high risk will be referred to a psychologist for additional support.

Models of psychological care have been previously described that stratify the depth and focus of care according to an expressed need by screening patients [62], and this approach could be translated and tested in prehabilitation settings. Care can then be stepped up as needed, when distress does not ameliorate or the patient’s clinical and/or psychosocial context changes [63]. Importantly, in low-intensity models such as this, accessibility needs to be at the center of the care model along with a consideration of implementation readiness. Finally, given the role of community and civil society in many cultural settings, articulation with community resources such as peer support is critical. A current ongoing randomized controlled trial uses this modality. In this study, which examines the impact exercise prehabilitation with or without psychological support, the research team has partnered with local cancer charities to deliver the psychological component of the intervention [64]. Results of this trial were due in 2022; however, at the time of writing it was paused due to the COVID-19 pandemic.

## Recommendations for Future Research and Clinical Practice

### Methodological Rigor

Some of the uncertainties in the current evidence base regarding the impact of psychological prehabilitation interventions can be overcome with appropriate attention to design, delivery, and reporting of future interventional studies. Researchers are encouraged to use the template for the intervention description and replication (TIDieR) checklist [65]. This will afford reviewers, fellow academics, and clinicians sufficient detail to replicate interventions and aid evidence synthesis.

### Measures and Timing

Consensus around a core outcome set to measure impact on psychological morbidity is also required for use in both unimodal psychological prehabilitation interventions as well as multimodal approaches. Agreement around optimal timing of assessments in relation to surgery and neoadjuvant/adjvant therapies is also warranted. Furthermore, a longer-term follow-up would allow investigation of the long-term impacts of these interventions on psychological health.

### Screening and Stratification

Recent prehabilitation guidance and position statements advocate for early psychological assessment and subsequent stratified support [60••, 66••]. However, there is no agreement on the most appropriate method/s of screening and stratification in the prehabilitation setting. Existing screening tools widely used in psycho-oncology, such as the NCCN Distress thermometer, GAD-7, PHQ-9, and Emotions Thermometer with established clinical cut-offs for severity, provide a useful indication of those who may be at risk of poorer psychological outcomes and in need of additional support [15]. However, supportive infrastructure, processes, and an appropriate workforce must be in place to enable more detailed assessment when indicated, and onward referral for those with complex needs. The existing clinical provision of such services varies between and within nations. Lessons can be learned from established models of care in Australia, for example, with an evidence-based clinical practice pathway for the screening and management of anxiety and depression in patients with cancer [62, 67]. This pathway guides staffing, timing, and intervention content to facilitate implementation into routine practice, and thereby ensure timely identification and care of patients with anxiety and depression. It is also vital that psychological assessment is ongoing across the cancer continuum to ensure appropriate support as patient needs change throughout treatment and recovery.

### Intervention Delivery

What constitutes an appropriate and effective intervention at the different levels of patient need and who is best placed to deliver them in the prehabilitation setting is largely untested [62]. The Principles and Guidance for Prehabilitation Within the Management and Support of People with Cancer [60••] recommends a triage system that includes universal, targeted, and specialist levels of prehabilitation. This strategy proposes that all healthcare professionals can be equipped to provide “universal” psychological support. “Targeted” interventions, for example, supporting problem-solving and solution-focused therapies, require additional expertise, and “specialist” support should be provided by those with specialist training, such as psychologists, specialist nurses, and relevant allied health workers. However, multiple infrastructure, organizational, and economic challenges exist to implement this guidance: implementation of psychosocial healthcare interventions is inherently complex necessitating specialist staffing, training, and resources in real-world practice settings [68]. For example, healthcare professionals who might be expected to provide “universal” support in clinical settings, such as healthcare assistants, may lack the confidence and knowledge of how best to support the psychological needs of patients and how to identify those in need of additional support and often do not have suitable referral pathways. Evidence-based, accessible, and tailored educational programs for health professionals at all levels can improve skills and confidence in delivering appropriate interventions, and consequently improve care. Furthermore, clinical services should articulate with community-based cancer and welfare organizations, including the charity sector and civil societies, who historically have led the provision of psychosocial care after cancer [69, 70].

### Patient Characteristics

As well as screening for psychological status, those developing and delivering prehabilitation programs and services should consider demographic and clinical characteristics that put patients at risk of poorer outcomes. These include lower socioeconomic status, education and health literacy, younger age, advanced-stage disease, greater treatment morbidities, and being single or unpartnered [13, 71]; patients typically under-represented in clinical trials.

## Conclusions

Provision of psychological support is advocated throughout the cancer continuum. Identifying those patients at risk of poorer outcomes as close to diagnosis as possible and intervening early through prehabilitation programs has the potential to improve psychological health, to prevent longer-term psychological morbidity, and to improve treatment outcomes. Such an approach aligns with the decade-old principles that psychological care is critical to ensure high-quality cancer care [15]. Existing evidence for the impact of prehabilitation interventions is limited due to methodological limitations within the literature. We encourage those designing and reporting new clinical trials to provide sufficient information to replicate their intervention. Attention should also be given to ensure new programs have the potential to be implemented within clinical practice with consideration for designing and testing care that is accessible and has the potential for large-scale implementation.

## Supplementary Information

ESM 1 (PDF 273 kb)
