# Supplementary material for: Psychological Interventions Prior to Cancer Surgery: a Review of Reviews
Source: Curr Anesthesiol Rep. 2022 Jan 31;12(1):78–87. doi: 10.1007/s40140-021-00505-x (PMC8801554; doi:10.1007/s40140-021-00505-x)
Supplement: Supplementary file 1 — (PDF 273 kb) [file 40140_2021_505_MOESM1_ESM.pdf]

Table S1: Overview of psychological intervention types [54]

		Education				Cognitive-behavioral					Relaxation						Supportive Counseling				
	**Number of papers with psychological intervention / Total papers**	**Education**	**Psychoeducation**	**Information provision**	**Stress management training**	**CBT (not specified)**	**Modified sleep restriction**	**Stress management (CBT orientation)**	**Problem-solving**	**Coping skills**	**Anxiety reduction**	**Guided imagery**	**Relaxation training**	**Progressive muscle relaxation**	**Breathing techniques**	**Meditation**	**Psychotherapy**	**Health professional discussion**	**Counseling (not specified)**	**Supportive expressive therapy**	**Supportive attention**
Boereboom et al. (2016)	2/8										✓										
Bolshinsky et al. (2018)	2/20										✓										
Bruns et al. (2016)	2/6										✓										
Chen et al. (2018)	8/8					✓	✓	✓	✓	✓		✓	✓	✓	✓		✓			✓	
Chou et al. (2018)	4/12		✓		✓					✓	✓	✓	✓								
Daniels et al. (2020)	6/52		✓	✓						✓	✓	✓	✓				✓				
Fitzgerald et al. (2016)	21/21			✓															✓		
Garcia et al. (2016)	2/14	✓											✓								
Hijazi et al. (2017)	2/9										✓										
Pouwels et al. (2015)	2/11														✓						
Rosero et al. (2019)	2/10		✓		✓																
Schneider et al. (2020)	5/15		✓								✓						✓				
Steffens et al. (2018)	2/17														✓						
Treanor et al. (2018)	6/18		✓		✓								✓				✓				✓
Tsimopoulou et al. (2015)	7/7	✓	✓		✓				✓	✓	✓	✓	✓			✓	✓	✓			✓
Vermillion et al. (2018)	2/9										✓										

Education: information or education about cancer, treatment, and/or physical side effects; ***Psycho-education***: information or education about emotional impact of cancer and stress management, excludes cognitive-behavioral approaches. **Cognitive-behavioural**: working with cognitions, challenging negative thoughts, refocusing thoughts onto positive; goal-setting and problem-solving or behavioral maintenance. **Supportive counseling**: counseling or therapy offered as part of the intervention, excludes cognitive-behavioral approaches; ***Health professional discussion***: discussion with a health professional (excludes counseling/psychotherapy, routine/standard care). *Abbreviations*: CBT, cognitive behavioral therapy
